# Dengue Infection Susceptibility of Five *Aedes aegypti* Populations from Manaus (Brazil) after Challenge with Virus Serotypes 1–4

**DOI:** 10.3390/v14010020

**Published:** 2021-12-23

**Authors:** Bárbara Aparecida Chaves, Raquel Soares Maia Godoy, Thaís Bonifácio Campolina, Ademir Bentes Vieira Júnior, Andréia da Costa Paz, Evelyn Beatriz da Costa Vaz, Breno Mello Silva, Rêgila Mello Nascimento, Maria das Graças Vale Barbosa Guerra, Marcus Vinicius Guimarães Lacerda, Wuelton Marcelo Monteiro, Nágila Francinete Costa Secundino, Paulo Filemon Paolucci Pimenta

**Affiliations:** 1 Fundação de Medicina Tropical Heitor Vieira Dourado, Manaus 69040-000, Brazil; bachaves89@gmail.com (B.A.C.); ademir.bentes.jr@gmail.com (A.B.V.J.); andreia.cpaz@gmail.com (A.d.C.P.); evelyn.beatrizcv@gmail.com (E.B.d.C.V.); regilamn@gmail.com (R.M.N.); barbosamgvale@gmail.com (M.d.G.V.B.G.); marcuslacerda.br@gmail.com (M.V.G.L.); wueltonmm@gmail.com (W.M.M.); 2 Programa de Pós-Graduação em Medicina Tropical, Universidade do Estado do Amazonas, Manaus 69850-000, Brazil; secundinon@gmail.com; 3 Instituto de Pesquisas René Rachou, Fiocruz, Belo Horizonte 30190-002, Brazil; quel_maia1@hotmail.com (R.S.M.G.); thais_campolina@hotmail.com (T.B.C.); 4 Departamento de Ciências Biológicas, Universidade Federal de Ouro Preto, Ouro Preto 35400-000, Brazil; breno@ufop.edu.br

**Keywords:** DENV, antiviral response, mosquito population, vector-borne disease, vector competence, infection rate, viral load

## Abstract

The successful spread and maintenance of the dengue virus (DENV) in mosquito vectors depends on their viral infection susceptibility, and parameters related to vector competence are the most valuable for measuring the risk of viral transmission by mosquitoes. These parameters may vary according to the viral serotype in circulation and in accordance with the geographic origin of the mosquito population that is being assessed. In this study, we investigated the effect of DENV serotypes (1–4) with regards to the infection susceptibility of five Brazilian *Ae. aegypti* populations from Manaus, the capital of the state of Amazonas, Brazil. Mosquitoes were challenged by oral infection with the DENV serotypes and then tested for the presence of the arbovirus using quantitative PCR at 14 days post-infection, which is the time point that corresponds to the extrinsic incubation period of *Ae. aegypti* when reared at 28 °C. Thus, we were able to determine the infection patterns for DENV-1, -2, -3 and -4 in the mosquito populations. The mosquitoes had both interpopulation and inter-serotype variation in their viral susceptibilities. All DENV serotypes showed a similar tendency to accumulate in the body in a greater amount than in the head/salivary gland (head/SG), which does not occur with other flaviviruses. For DENV-1, DENV-3, and DENV-4, the body viral load varied among populations, but the head/SG viral loads were similar. Differently for DENV-2, both body and head/SG viral loads varied among populations. As the lack of phenotypic homogeneity represents one of the most important reasons for the long-term fight against dengue incidence, we expect that this study will help us to understand the dynamics of the infection patterns that are triggered by the distinct serotypes of DENV in mosquitoes.

## 1. Introduction

Dengue is an arthropod-borne disease that affects many people globally and causes about 300–400 million cases/year [1]. Today, the disease affects the populations of more than 100 countries in Asia, the Americas, Africa, the Caribbean, and the Pacific. Despite all the control strategies, the dengue virus (DENV) continues to spread in many regions due to highly competent mosquito vectors [2]. The primary vector of dengue in the New World is *Aedes (Stegomyia) aegypti*, which circulates in great abundance in many locations [3,4]. The Pan American Health Organization [5] reported 2,326,115 and 1,067,849 cases of dengue in the Americas in 2020 and 2021, respectively, with 63.1 and 80.1% of these cases occurring in Brazil [6].

In all, four phylogenetically related but antigenically distinct dengue virus serotypes (DENV-1, DENV-2, DENV-3, and DENV-4) currently circulate around the globe. This co-circulation of different serotypes is epidemiologically important since antibodies derived from an initial DENV infection may enhance the severity of a secondary infection by a heterologous serotype [7,8,9]. Presently, all four DENV serotypes are in circulation in the Americas (PAHO, 2021). In Brazil, although dengue has been present since the 1600s [10], the first DENV serotypes identified were DENV-1 and DENV-2 in 1990s, followed by DENV-3 in 2000 [11]. These three serotypes were detected every year until 2008, when DENV-4 was introduced in the country’s northern region [11,12,13,14]. In 2020, all four DENV serotypes were detected in Brazil [6,15]. In Manaus, the capital city of the Amazonas state, all of the DENV serotypes have already been detected [16].

Many factors, such as the immunity of the human host, vector density, vector competence, and environmental conditions, influence the dispersal of DENV [17,18,19], and the best method for controlling the disease is to fight the mosquito vector. Several studies have focused on understanding the vector-virus interface of DENV in *Ae. aegypti* up until the point when it is transmitted to humans. A mosquito’s susceptibility or resistance to arboviruses like DENV is associated with its anatomical barriers, such as the midgut and the salivary gland, and these determine vector competence [20]. The assessment of vector-competence-related parameters in *Ae. aegypti* populations have been long used to monitor areas at risk of dengue and to prevent a resurgence of dengue outbreaks [21,22,23,24].

The susceptibility of *Ae. aegypti* to DENV infections varies among populations from different countries, states of the same country, and even among separate locations within the same city [22,25,26,27]. Susceptibility to arbovirus infection [28] and the efficiency of disease transmission [29] depend on the genetic and geographical background of mosquito populations [30]. In addition, a mosquito’s susceptibility to arboviruses is also dependent on the DENV serotype/genome involved in the infection [24,31,32,33].

Some studies of the population genetics of *Ae. aegypti* have found genetic divergence in distinct mosquito populations within a city [34,35,36,37,38,39]. Two studies compared the genome of *Ae. aegypti* populations from Manaus and showed a small genetic divergence [40,41]. As a result, we decided to investigate the level of variation in infection susceptibility presented by regionally separated *Ae. aegypti* mosquitoes in Manaus. Since the virus genome is one of the factors that causes differences in infection patterns, we challenged mosquito populations with the four DENV-serotypes to attest to whether their infection parameters would tend to similarity.

In the present study, we analyzed the infection rate, disseminated infection rate, assumed vector competence, and viral loads of five geographically distinct field populations of *Ae. aegypti* of a Brazilian city after challenging them with the DENV serotypes 1–4. Mosquito eggs were collected in field sites of distinct regions in the city of Manaus, and the adult females derived from them at F3–F4 generations were used in experiments. The *Ae. aegypti* infection patterns related to all DENV serotypes were characterized and compared, and their relationship with the mosquito’s anatomical barriers are discussed in the context of interpopulation and inter-serotype variability.

## 2. Materials and Methods

### 2.1. Study Area and Mosquito Collection

The study was conducted in Manaus, the capital of Amazonas state (03°06′07″ S, 60°01′30″ W), which is the largest and the most populous metropolitan city in the northern region of Brazil (11,401,092 km^2^ and 2,219,580 inhabitants). The city is divided into six health districts: northern, southern, eastern, western, south-central and west-central [42]. The last two districts have comparatively small geographic areas, and, in this study, to avoid inconsistency in the size of the collection areas, they were combined and named the central district.

In order to collect eggs laid by *Ae. aegypti* females, 20 ovitraps were positioned for 5 days in several urbanized locations of each district, considering previous knowledge of the vector presence. The eggs of these 20 ovitraps (30 to 200 eggs per ovitrap) were mixed and allowed to hatch, then reared until the adult stage. The adult *Ae. aegypti* were separated, and each collection was named according to the health district of origin. The mosquitoes were kept in an insectary at a controlled temperature of 28 °C, 80% relative humidity, and 12 h/12 h light-dark photoperiod. The *Ae. aegypti* were raised for 3–4 generations until there were enough adult specimens to be used in the experimental infections.

Samples of first-generation adults (parental generation) from the collection in each district were confirmed to be negative for natural DENV infection using real-time PCR (qPCR), as described below. This examination of possible natural DENV infection of the samples was necessary since this study was developed with adult mosquitoes derived from field-collected eggs from endemic areas.

### 2.2. Virus Culture

*Ae. aegypti* from each city district were simultaneously challenged with isolated strains of DENV-1 (KP188540), DENV-2 (KP188569), DENV-3 (BR74886/02), and DENV-4 (KP188566). These strains were kindly provided by the Virology Research Laboratory at the São José do Rio Preto College of Medicine, São Paulo. Virus titers of 1 × 10^5^ plaque-forming units per mL (titration performed according TCID_50_ method) [43] were maintained and multiplied in C6/36 cell culture supernatants using Leibowtiz (L-15) medium supplemented with 5 μg/mL amphotericin B, 200 U/mL penicillin-streptomycin and inactivated fetal bovine serum [23].

### 2.3. Experimental Infection with the DENV Serotypes

A total of 150 females (n = 150) of each population were placed in separate cages. For the experimental infections, glass mosquito feeders were filled with mouse (*Mus musculus*) blood (two-thirds) mixed with each of the DENV serotypes (one third, viral suspension at 3 × 10^5^ PFU/mL) and offered to the mosquitoes for 2 h, as described elsewhere [22,27,44]. After the blood meal, all fully engorged females were separated and maintained with 10% glucose solution *ad libitum* under insectary conditions until the 14th day post infection (dpi), which is when the extrinsic incubation period (EIP) is complete and the *Ae. aegypti* become infectious [45].

### 2.4. Extraction and Quantification of Viral RNA Using Real-Time PCR (RT-qPCR)

After the completion of the EIP (14 dpi), 40 *Ae. aegypti* that were experimentally infected with the DENV serotypes from each health district were randomly separated into 5 groups. These mosquitoes were anesthetized on ice and dissected under a stereoscope. Their bodies and heads with the attached salivary gland (heads/SG) were individualized and transferred to separate microtubes. According to the manufacturer’s instructions, the viral RNA was extracted from each mosquito sample using the QIAamp**^®^** viral RNA mini kit (Qiagen, Germantown, MD, USA) and subsequently stored at −70 °C.

RT-qPCR reactions were performed in an ABI Prism 7500 Fast Real-time PCR machine (Applied Biosystems, Waltham, MA, USA) using the Power SYBR**^®^** Green RNA-to-Ct 1-step detection system (Applied Biosystems). A pair of generic primers was used to amplify all DENV, forward (5′-AGGACYAGAGGTTAGAGGAGA-3′) and reverse (5′-CGYTCTGTGCCTGGAWTGAT-3′) [46]. The viral RNA extracted from the bodies and heads/SG of the *Ae. aegypti* were tested, and testing of all samples was performed in duplicate, with positive and negative controls. The negative controls were the mosquitoes from the health districts that had been submitted to a non-infective blood meal. The positive controls were *Ae. aegypti* from a colonized PP strain that are used routinely and always present viral detection after an infective blood meal [47]. The results were considered positive, according to the melting curve (78.6 ± 0.5 °C) and Ct < 35, following the rigorous criteria from the literature for the minimum information for publication of quantitative real-time PCR experiments (MIQE) (Bennett et al., 2002). For measurement of viral load, we compared the ΔΔCt value of each sample with the values from a reference curve of samples containing 3 × 10^1^ to 3 × 10^7^ copies of a linearized plasmid with the DENV genome fragment corresponding to the non-coded portion of the 3′ region of the RNA (nt10576 to nt10683). This protocol has been previously used in other studies for estimating viral load and comparing different experimental conditions [22,26,48].

### 2.5. Infection Rate, Disseminated Infection Rate, and Vector Competence

Infection rate (IR), disseminated infection rate (DIR), and vector competence (VC) were determined for the five *Ae. aegypti* populations of Manaus that were challenged with each of the four DENV serotypes. IR was calculated as the proportion of infected mosquitoes in relation to the total number of tested mosquitoes (n = 40), and DIR was the proportion of infected mosquito heads/SG in relation to the number of infected mosquitoes. VC was the proportion of infected mosquito heads/SG in relation to the number of tested mosquitoes. Therefore, the mosquitoes were not assayed for salivary-gland infection or actual virus transmission. Instead, VC was assumed to be the same as the rate of virus dissemination to the head/SG tissues, following the same method used elsewhere [26,27,49,50].

### 2.6. Statistical Analyses

For comparison of IR, DIR, and VC sets between DENV-1, DENV-2, DENV-3, and DENV-4, two-way ANOVA (multiple comparisons) and Tukey’s range test were used. Mann-Whitney U tests were used to evaluate significance between viral-load medians in the bodies and heads/SG for each mosquito population infected with all DENV serotypes, and viral-load medians in the bodies and heads/SG for the total population (considering the sum of all populations) infected with all DENV serotypes. Evaluations of the body viral loads and head/SG viral loads among all populations and among all serotypes were performed using Kruskal-Wallis one-way ANOVA tests. All statistical analyses were performed using GraphPad Prism, version 7.00 (GraphPad, San Diego, CA, USA), and *p* values ≤ 0.05 were considered statistically significant.

## 3. Results

### 3.1. Vector Competence of Ae. aegypti Populations in Relation to DENV Serotypes

**Figure 1:**
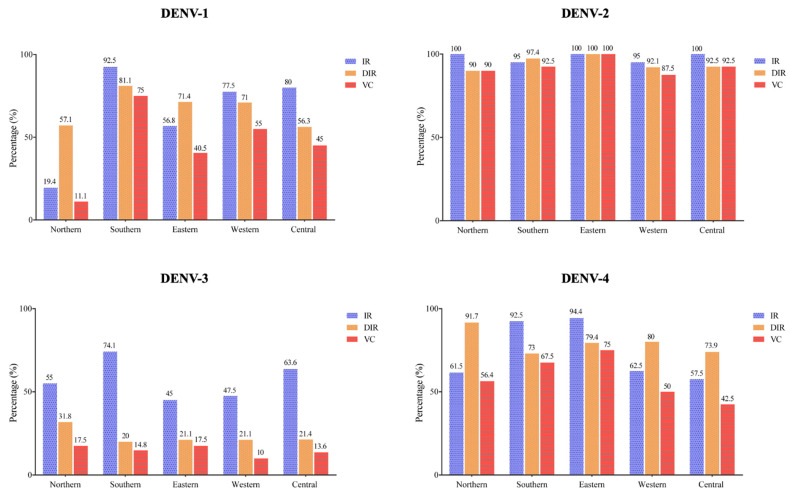
Infection rate (IR), disseminated infection rate (DIR), and assumed vector competence (VC) of *Ae. aegypti* populations from Manaus (Brazil) for DENV serotypes 1–4.

**Figure 2:**
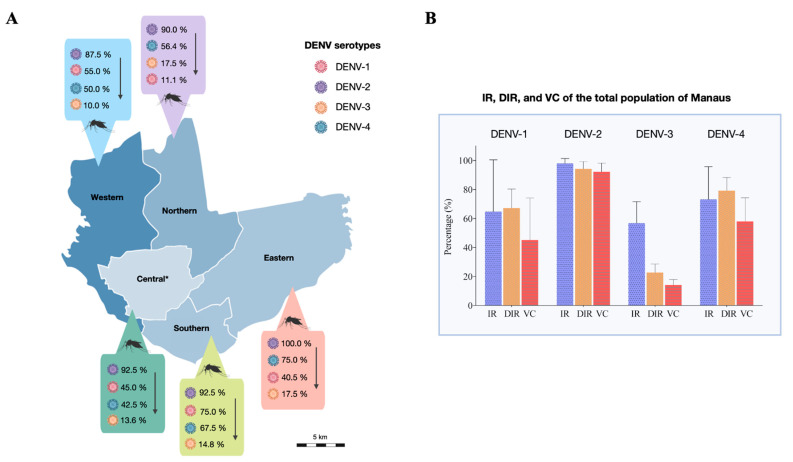
Variability in the infection rate (IR), disseminated infection rate (DIR) and the assumed vector competence (VC) of the *Ae. aegypti* populations for DENV serotypes 1–4. (**A**): Map of the urban area of Manaus showing the mosquito VC for each population (derived from distinct geographic districts), considering the order of decreasing values. The south-central and west-central districts were combined and named the central district to avoid inconsistency in the size of the collection areas. (**B**): IR, DIR, and VC averages of all populations show similarity in infection patterns between DENV-1 and DENV-4. The error bars on the graph are the standard deviations. The standard deviations of the IRs, DIRs, and VCs, respectively, were 28.7%, 10.6%, and 23.3% for DENV-1; 2.7%, 4.1%, and 4.7% for DENV-2; 12%, 4.9%, and 3.1% for DENV-3; and 18.2%, 7.5%, and 13.1% for DENV-4.

All of the *Ae. aegypti* populations from the five health districts of Manaus had individuals that were susceptible to infection with the four DENV serotypes. Infection rate (IR), disseminated infection rate (DIR), and vector competence (VC) showed differences for all DENV serotypes among the five populations (Figure 1 and Figure 2A):

DENV-1 infection. IR ranged from 19.4% (northern) to 92.5% (southern) and DIR ranged from 56.3% (Central) to 81.1% (Southern). The lowest VC was 11.1%, from the northern population, and the highest VC was 75%, from the southern population.

DENV-2 infection. The IR was 95% (Southern and Western) or 100% (Northern, Eastern and Central), and the DIR ranged from 92.1% (Western) to 100% (Eastern). The lowest VC was 87.5%, from the Western population, and the highest VC was 100%, from the Eastern population.

DENV-3 infection. IR ranged from 45% (eastern) to 74.1% (southern), and DIR ranged from 20% (southern) to 31.8% (northern). The lowest VC was 10%, from the western population, and the highest VC was 17.5%, from the northern and eastern populations.

DENV-4 infection. IR ranged from 57.5% (central) to 94.4% (eastern), and DIR ranged from 73% (southern) to 91.7% (northern). The lowest VC was 42.5%, from the central population, and the highest VC was 75%, from the eastern population.

Considering the infection response of each *Ae. aegypti* population to the DENV serotypes, the western, southern and central populations were more competent at transmitting DENV-2, which was followed by DENV-1, DENV-4, and DENV-3. The northern population was more competent at transmitting DENV-2, followed by DENV-4, DENV-3, and DENV-1. For the eastern population, greater competence was observed for DENV-2, followed by DENV-4, DENV-1, and DENV-3 (Figure 2A).

The mean IRs for DENV-1, DENV-2, DENV-3, and DENV-4, respectively, were 65.3, 98, 57, and 81.4%. Is this same order, the mean DIRs were 69.6, 94.4, 23.1, and 79.6%; and the mean VCs were 45.32, 92.5, 14.68, and 58.28%. To obtain the estimate of DENV infection susceptibility for all of the *Ae. aegypti* mosquitoes from Manaus, we combined the results of all *Ae. aegypti* populations, assuming that they represent the natural mosquito diversity in the total territory of the city. The means of the IRs, DIRs, and VCs of all mosquito populations among the DENV serotypes were compared, and it was observed that the DENV-1 results were similar to those of DENV-4; all the other possible comparisons were statistically distinct. The IR and DIR means were similar to each other for infection with DENV-1, DENV-2, and DENV-4; however, for DENV-3, the DIR was lower than the IR (Figure 2B).

### 3.2. Viral Quantification of the Ae. aegypti Populations

**Figure 3:**
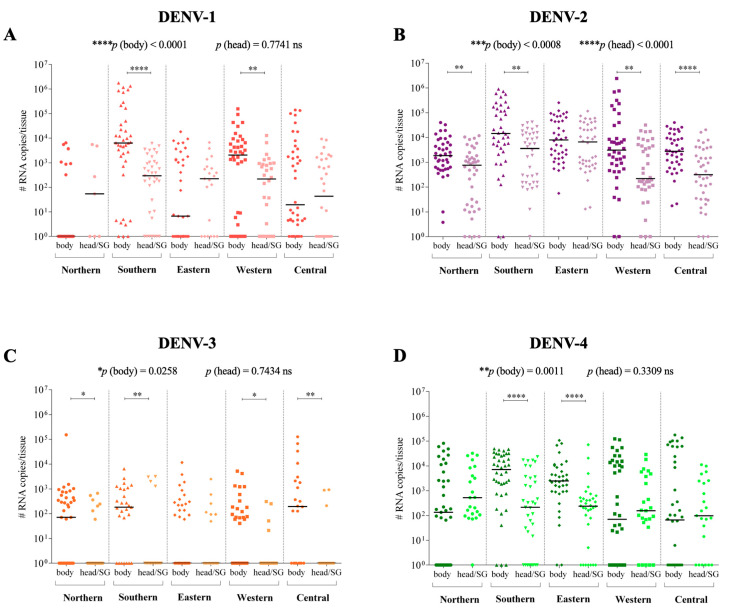
Body and head/SG viral loads of five *Ae. aegypti* populations from different districts of the municipality of Manaus, Brazil after infection with DENV-1 (**A**), DENV-2 (**B**), DENV-3 (**C**), and DENV-4 (**D**). *p* values > 0.05 [non-significant (ns)] are not represented. *p* values ≤ 0.05, ≤0.01, ≤0.001, and ≤0.0001 are summarized with one (*), two (**), three (***), and four (****) asterisks, respectively.

Viral quantification of the *Ae. aegypti* populations, which compared the infections with the same DENV serotypes, showed significant dissimilarities (Figure 3):

DENV-1 infection. The head/SG viral loads in western and southern populations were lower than the body viral loads (*p* = 0.005 and *p* < 0.0001, respectively). However, the body and head/SG viral loads were similar for the northern, eastern, and central populations (*p* = 0.0579, *p* = 0.7632, and *p* = 0.1394, respectively). The body viral loads varied among themselves (*p* < 0.001), but the head/SG viral loads were similar to one another (*p* = 0.7741) (Figure 3A).

DENV-2 infection. The head/SG viral loads in northern, western, southern, and central populations were lower than the body viral loads (*p* = 0.0047, *p* = 0.0056, *p* = 0.0010, and *p* < 0.0001, respectively). The body and head/SG viral loads in the eastern population were analogous (*p* = 0.1504). Both the body and head/SG viral loads varied among themselves (*p* = 0.0008 and *p* < 0.0001, respectively) (Figure 3B).

DENV-3 infection. The head/SG viral loads in the northern, western, southern, and central populations were lower than the body viral loads (*p* = 0.0337, *p* = 0.0356, *p* = 0.0086, and *p* = 0.0087, respectively). The body and head/SG viral loads in the eastern population were similar (*p* = 0.5218). The body viral loads varied among themselves (*p* = 0.0258), but the head/SG viral loads were similar to one another (*p* = 0.7434) (Figure 3C).

DENV-4 infection. The head/SG viral loads in the eastern and southern populations were lower than body viral loads (both showed *p* < 0.0001). The viral loads between the body and head/SG in the northern, western and central populations were similar (*p* = 0.1279, *p* = 0.9431, and *p* = 0.8673, respectively). The body viral loads varied among themselves (*p* = 0.0011), but the head/SG viral loads were similar to one another (*p* = 0.3309) (Figure 3D).

**Figure 4:**
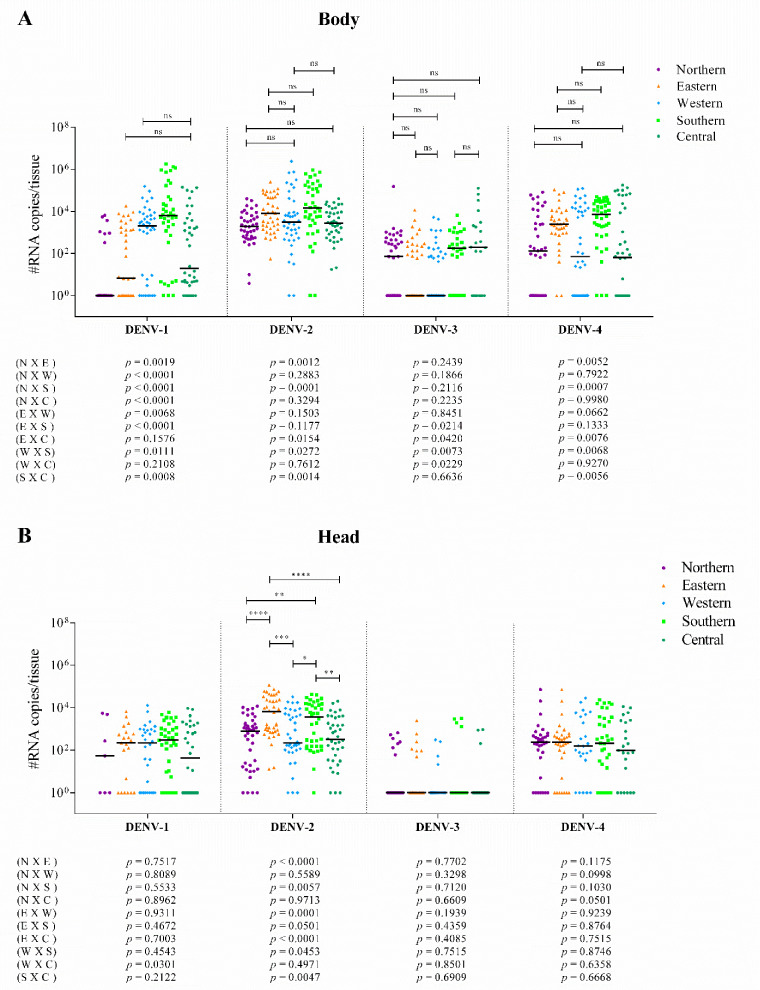
Interpopulation variability in the body (**A**) and head/SG (**B**) viral-load patterns of *Ae. aegypti* in the northern, southern, eastern, western, and central populations (different colored symbols). Each bar represents a median viral load in the data. In (**A**), we represent only the non-significant results (more results were statistically distinct) to simplify the demonstration and facilitate comparisons. In (**B**), we represent only the significant results. All *p* values were listed for the 10 possible comparisons among the mosquito populations to each DENV serotype. N = northern; E = eastern; W = western; S = southern; C = central. *p* values > 0.05 (non-significant) are represented as “ns”. *p* values ≤ 0.05, ≤0.01, ≤0.001, and ≤0.0001 are summarized with one (*), two (**), three (***), and four (****) asterisks, respectively.

For DENV-1, DENV-3, and DENV-4, the body viral load varied among the populations, but the head/SG viral loads were similar. Differently for DENV-2, both body and head/SG viral loads varied among the populations. These patterns of variability can be visualized in Figure 3 through the statistical representation of the general comparison among the populations [*p* (body) and *p* (head)]; and in more detail in Figure 4, in which the comparisons were made one by one. The pattern of interpopulation variability of the body viral loads showed similarity between DENV-2 and DENV-4 infections; however, for the head viral load, the pattern differed (Figure 4).

**Figure 5:**
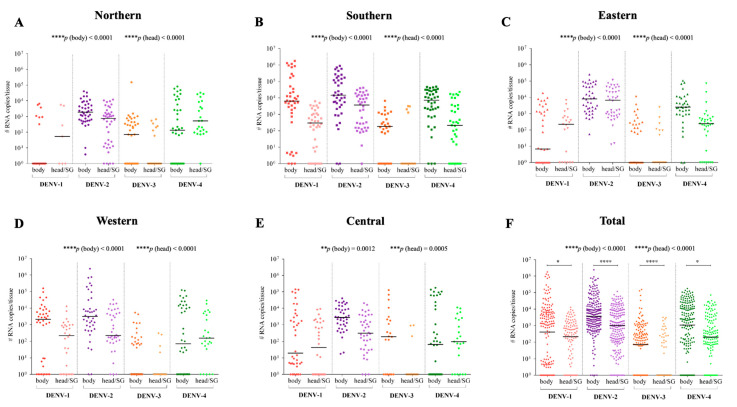
Body and head/SG viral loads of *Ae. aegypti* for the four DENV serotypes in the northern (**A**), southern (**B**), eastern (**C**), western (**D**), and central (**E**) populations; and the overall body and head/SG viral loads (**F**). *p* values > 0.05 [non-significant (ns)] are not represented. *p* values ≤ 0.05, ≤0.01, ≤0.001, and ≤0.0001 are summarized with one (*), two (**), three (***), and four (****) asterisks, respectively.

**Figure 6:**
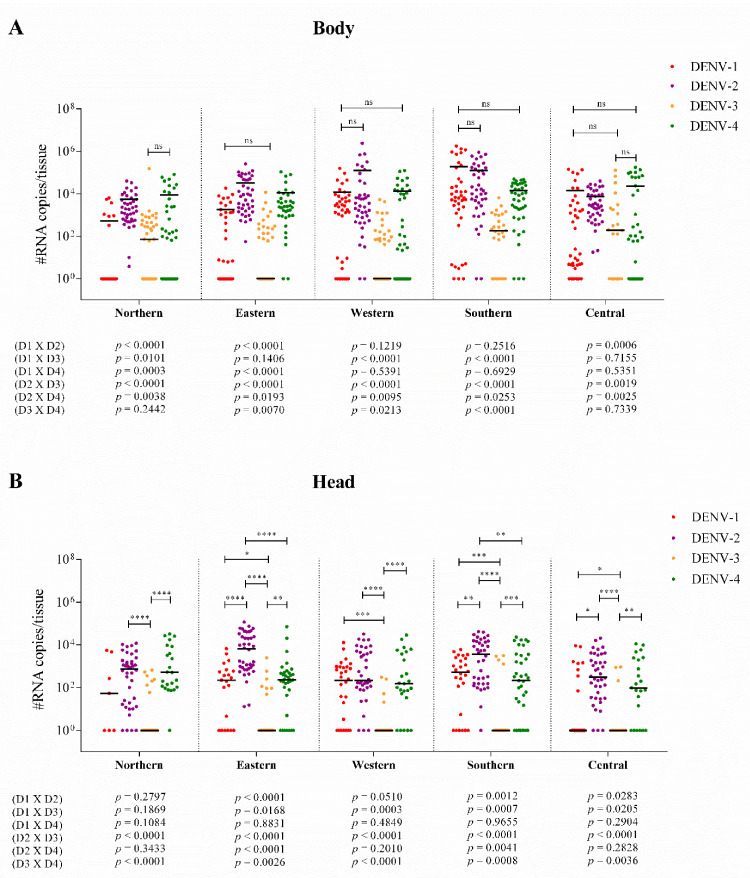
Inter-serotype variability of the patterns of body (**A**) and head/SG (**B**) viral loads for DENV serotypes 1–4 (different colored symbols) in each of the five *Ae. aegypti* populations. Each bar in the figures represents a median viral load in the data. In (**A**), we represent only the non-significant results (more results were statistically distinct) to simplify the demonstration and facilitate comparisons. In (**B**), only the significant results are represented. All *p* values are listed for the six possible comparisons among the DENV serotypes to each population. D1 = DENV-1; D2 = DENV-2; D3 = DENV-3; D4 = DENV-4. *p* values > 0.05 (non-significant) are represented as “ns”. *p* values ≤ 0.05, ≤0.01, ≤0.001, and ≤0.0001 are summarized with one (*), two (**), three (***), and four (****) asterisks, respectively.

Considering the variability in viral load among the DENV serotypes, both body viral loads and head/SG viral loads vary considerably (Figure 5A–E). When we combine the results of all *Ae. aegypti* populations, assuming that they represent the natural diversity of mosquitoes in the whole of the territory of the city, viral load also shows substantial variability among the serotypes in both body and head/SG tissues. In addition, body viral load is higher than head/SG viral load for all DENV serotypes (Figure 5F). The statistical representation of the general comparison among the serotypes [*p* (body) and *p* (head)] is shown in Figure 5, and the details of this analysis, with one-by-one comparison, are represented in Figure 6. The pattern of inter-serotype variability of body viral loads shows similarity between the western and southern populations; however, for head/SG viral load, the pattern differs. The pattern seen for the eastern and southern populations is similar for the head/SG viral loads, but differs for body viral loads (Figure 6).

In general, the five *Ae. aegypti* populations had distinct viral responses when considering all the parameters assessed and the four DENV serotypes. No association was detected among neighboring mosquito populations or between any of the mosquito populations from Manaus.

## 4. Discussion

This study shows that Brazilian *Ae. aegypti* mosquitoes from Manaus have interpopulation variations in response to DENV serotype infections. Specific environmental conditions associated with the level and period of exposure to different pathogens, such as the temperature, nutrition, and breeding water of mosquitoes while in the immature stages, are factors that may drive the capability of invertebrate vectors to host and spread vector-borne pathogens in the short and long term [22,26,27,47]. The distinct selection pressures to which the vectors are subjected tend to modulate their evolution and favor genetic divergence. In turn, distinct vector genomes influence the resistance and/or susceptibility to arboviral invasion, as well as viral dissemination to salivary glands after an infected blood meal. Essentially, the vector’s immunity, microbiota composition, mechanisms of midgut cell regeneration, and intrinsic physical barriers are aspects that may vary among mosquito populations as a result of genetic divergence [17,47,51]. Low to high levels of genetic divergence have been detected in the neighborhoods of cities where *Ae. aegypti* is present [34,35,37,38], including those of Manaus [40,41].

The lack of association among *Ae. aegypti* populations regarding the parameters assessed for susceptibility to DENV 1–4 can be related to the distinct geographical characteristics presented by the five regions of origin of the mosquitoes. In general, the entire territory has a hot and humid climate, with significant rainfall almost every month of the year [52]. The western and the eastern regions possess the largest green areas compared to other regions, while hypsometry and the geothermal profiles differ among the regions [53,54]. The central, northern, and southern regions also have distinct urbanization and geothermal profiles [53]. These geographical differences among the regions of Manaus may indirectly drive the discrepancy in DENV susceptibilities of the mosquito populations by reflecting the distinct selective pressures to which they are subjected. The level of genetic divergence that Da Costa-Fraga et al. (2003) [40] and Santos et al. (2011) [41] found for the regionally separated *Ae. aegypti* populations from Manaus was small, indicating that gene flow in the city may be high, thus alleviating the genetic structure. In the present year (2021), we do not know whether the status of the genetic divergence among the mosquitos has changed so that we can make a more up-to-date inference. However, as we have shown in the present work, the differences presented by the *Ae. aegypti* populations are already capable of affecting susceptibility to DENV infection.

The variability observed in the infection patterns of the same mosquito population for the distinct serotypes may be mainly attributed to the influence of the genetic divergence of the viral genomes. An important point to be considered is that there are DENV lineages of the same serotype that present lower levels of genetic divergence compared to that which exists among distinct serotypes [55]. Therefore, we can use our findings to represent the variability among serotypes, although we cannot state that all lineages would behave identically. In addition, to better represent virus-vector interactions in the field, all DENV lineages that we used are strains that circulate in Brazil. The genetic divergences of these four DENV serotypes can be seen by accessing their genomes on the NCBI Database [accession numbers: KP188540 (DENV-1), KP188569 (DENV-2), BR74886/02 (DENV-3), and KP188566 (DENV-4)].

The populations of *Ae. aegypti* in Manaus present distinct degrees of susceptibility to the four DENV serotypes. The five *Ae. aegypti* populations evaluated had different quantities of susceptible individuals that could transmit DENV-1, -2, -3 and -4. This shows possible virus-vector encounters in nature that could cause dengue outbreaks involving any of the four serotypes. Among the serotypes, the mosquitoes were more susceptible to DENV-2 since all the *Ae. aegypti* populations tested were highly susceptible to infection, and the viral loads were higher than those of the other serotypes. This result was expected since in Manaus and the rest of the country, most dengue cases are caused by DENV-2 [56,57].

Regarding the other serotypes, DENV-1 and DENV-4 come in second place according to mosquito susceptibility, with similar VC between them, followed by DENV-3, with the lowest VC and lowest viral loads of the four serotypes. Although we detected similarities between DENV-1 and DENV-4 infections in *Ae. aegypti* mosquitoes, it is important to note that their IR, DIR, VC, and viral loads differed in the context of the different vector populations. In practice, the similarities detected between DENV-1 and DENV-4 infections in *Ae. aegypti* mosquitoes led to a similar general risk estimate for causing dengue epidemics in Manaus. However, this does not mean that both serotypes produce similar infection responses in the vectors, as demonstrated by the regional results. This highlights the importance of monitoring *Ae. aegypti* mosquitoes in a more geographically restricted way in order to avoid potential erroneous estimates arising from generalizations.

Natural selection favors viral strains with higher virulence, which is characterized by higher infection and dissemination rates in mosquito vectors [58]. We may infer that for the mosquito populations tested, DENV-2 was and will probably continue to be favored by natural selection, contrasted with DENV-3, which tends to spread less among its *Ae. aegypti* vectors. Nonetheless, since the DENV serotypes are under constant and diverse selection pressures [58,59] and are also dependent on the susceptibility of human hosts to ensure evolutive success [60], this scenario is not static; it may change with the possible emergence of new positively selected mutations that occur in specific DENV serotype genotypes.

VC is defined as the intrinsic capacity of a mosquito to acquire and transmit a vector-borne pathogen. Conceptually, the assumed VC value is a product of the infection rate (IR) and the disseminated infection rate (DIR) (VC = IR × DIR), and these two rates are directly related to a vector’s susceptibility to the virus’s two main target organs: the midgut and the salivary gland (SG). IR indicates the virus’s ability to establish an initial infection in the vector’s midgut after ingesting an infective blood meal. Consequently, IR is related to the action of midgut infection barriers (MIBs), which is the first step in combating viral infection. MIBs may be related to incompatibilities between viruses and the epithelial midgut receptors, which prevent viral binding and entry into the mosquito’s cells [17]. Unlike IR, DIR shows the virus’s ability to disseminate from the midgut to the vector’s secondary organs and reach the SG. Therefore, the antiviral responses that affect DIR are the midgut escape barriers (MEBs). These responses prevent viruses inside the midgut from escaping and crossing the epithelium, the basal lamina, and/or tracheal system and reaching the hemocoel. Consequently, viruses inside the midgut are destroyed by the digestion of the blood meal or remain sequestered in the infected midgut cells. Salivary-gland infection barriers (SGIBs) prevent circulating viruses inside the hemocoel from invading, infecting, and replicating in the SG secretory cells [17,61]. Lastly, VC reflects the fluctuation of IR and DIR in a determined group or population of vectors since it indicates the vector’s capacity to be infected and maintain and transmit pathogens to humans in the next blood meal.

MIBs, MEBs, and SGIBs are physical barriers that may occur via innate immune responses of mosquitoes, such as the RNA interference (RNAi) pathway [62,63] and virion-tissue surface structure incompatibilities. They prevent the virus binding and/or traversal of target tissues that obstruct the pathogen’s life cycle [17]. The similarities between IR and DIR seen in *Ae. aegypti* infections (considering the total population) with the serotypes DENV-1, DENV-2, and DENV-4 suggest that the MIB, which is related to IR, and the MEB, which is related to DIR, influence the VC on an equal basis. Distinctly for DENV-3 infection, the MIB seems to be less effective in combating viral spread than the MEB.

Although the four DENV serotypes are phylogenetically related, they only share approximately 65% of their genomes, differing at similar levels to the diversity among flavivirus species [59,64]. These dissimilarities among the DENV serotypes lead to different efficiency levels in escaping the physical barriers and the immune response of the vectors. Notably, it has been demonstrated that the expression levels of immune factors in *Ae. aegypti*, such as the Toll receptor Spaetzle, its negative regulator Cactus, and the intracellular NF-kB-like factor, vary between DENV-1 and DENV-2 infections, thus implying the effects of inter-serotype variability [33]. Additionally, as viral invasion also depends on tissue-specific intrinsic factors (physical barriers), viruses from the same viral serotype/genotype may face distinct challenges when invading and replicating in target tissues/cells. This occurs because both general and virus-specific components are involved in virus-vector interactions [65,66]. Moreover, even though some of the defense mechanisms that are triggered are shared among the serotypes, serotype-specific responses strongly affect the vector’s capacity to deal with the viral infection. Characteristics such as pathogen-associated molecular patterns (PAMPs), pathogenicity, and/or cell/tissue tropisms may vary, depending on the specific DENV serotype [65,67].

Considering each of the *Ae. aegypti* populations of Manaus, viral loads (virus number) in the mosquito bodies were similar or superior to the head/SG loads for all DENV serotypes. However, if we consider all of the mosquito populations, the overall load was superior in the body than in the head/SG. Recent research using the same parameters to evaluate DENV-2 infections in nine *Ae. aegypti* populations in an endemic southeastern Brazilian city found similar results related to viral load [27]. This fact strengthens the hypothesis that DENV serotypes have tropisms for diverse mosquito tissues that replicate intensely and in large quantities in the mosquito hemocoel. A high viral load in the mosquito body may correspond to a striking viral strategy to ensure vertical transmission (ovary infection) [68], and it could ensure the maintenance of infection during the mosquito’s life span, thus improving viral transmission to vertebrate hosts. This condition of body viral load being superior to head/SG loads was not found in another two species of flavivirus infecting *Ae. aegypti*—the yellow fever virus (YFV) and Zika virus (ZIKV) [69]. YFV accumulates in greater amounts in the *Ae. aegypti* head/SG than in the body, even in coinfections with other arboviruses [69]. With regards to ZIKV, this virus accumulates in the head/SG tissues in detriment to the body in some *Ae. aegypti* populations, but it may also present a pattern similar to DENV in other populations. Therefore, although DENV, YFV, and ZIKV are phylogenetically related species of viruses, *Ae. aegypti* infection responses to them can vary significantly.

Interestingly, body viral loads show interpopulation variation for all DENV serotypes. Nonetheless, head/SG viral loads were similar in interpopulation variation for DENV-1, -3, and -4. Greater differences in viral efficiency in invasion of the midgut and secondary organs compared to viral efficiency in invasion and replication in the head tissues/SG may explain this result. As arboviruses possess tropisms in diverse body tissues, such as the ovaries, fat body, hemocytes, and muscle tissues [70,71], the source of variation is more prominent than that which occurs in the head/SG tissues, which have a smaller range of target tissues. However, as the literature lacks studies regarding DENV tropisms in vector tissues, this hypothesis cannot be strengthened by more consistent evidence.

In contrast to our results with the Brazilian vectors, *Ae. aegypti* from Cape Verde showed low vector competence for transmission of DENV-1 and DENV-4 and intermediate to high competence for transmission of DENV-2 and DENV-3 [72]. In another study, *Ae. aegypti* from the Caribbean were more competent at transmitting DENV-4 and less competent for the other three serotypes [23]. It seems there is no common tendency in *Ae. aegypti* susceptibility to infection according to distinct DENV serotypes. Therefore, mosquito susceptibility to viruses seems to be very particular to each pair of virus serotype/individual vector interaction. This specificity of coevolution between *Ae. aegypti* and viral serotypes and strains creates a problem when developing common strategies for combating dengue and other arthropod-borne diseases since some strategies may not work for all target vectors.

In conclusion, a particular mosquito population may exhibit variability in tissue-barrier efficiency for the DENV serotypes, and a particular viral serotype/genotype may encounter distinct infection barriers in different mosquito populations. It was observed that the mosquitoes had greater inter-serotype-related variations than interpopulation-related variations in terms of viral susceptibility, which indicates that the phenotypic discrepancies among the serotypes determined a wider range of infection variabilities than the phenotypic discrepancies among nearby mosquito populations in the city. The viruses of all DENV serotypes show a similar tendency to accumulate in the body in greater amounts than in the head/SG, which does not occur with other flaviviruses, such as YFV. Understanding the dynamics of infection-response variability in the vectors helps us to discover the reflex of the forces that shape DENV/vector coevolution in the triggering of antiviral defenses. Our results indicate the existence of a wide range of factors that, when combined, may generate a striking diversity of patterns of susceptibility to DENV infection, and this represents one of the most important reasons for the long-term fight against dengue incidence. Further studies focusing on the action of the distinct proteins coded by the DENV serotypes on the performance of the mosquito infection barriers, as well as studies analyzing which phenotypic characteristics are determined by the antiviral defense-related alleles of *Ae. aegypti*, could improve our understanding of infection-barrier functions in these mosquitoes.

## References

[1] Bhatt S., Gething P.W., Brady O.J., Messina J.P., Farlow A.W., Moyes C.L., Drake J.M., Brownstein J.S., Hoen A.G., Sankoh O. (2013). The global distribution and burden of dengue. Nature.

[2] ECDC 2020 Dengue Worldwide Overview. https://www.ecdc.europa.eu/en/dengue-monthly.

[3] Kraemer M.U., Sinka M.E., Duda K.A., Mylne A.Q., Shearer F.M., Barker C.M., Moore C.G., Carvalho R.G., Coelho G.E., Bortel W.V. (2015). The global distribution of the arbovirus vectors *Aedes aegypti* and *Ae. albopictus*. eLife.

[4] Kamal M., Kenawy M.A., Rady M.H., Khaled A.S., Samy A.M. (2018). Mapping the global potential distributions of two arboviral vectors *Aedes aegypti* and *Ae. albopictus* under changing climate. PLoS ONE.

[5] PAHO/WHO (2021). Number of Reported Cases of Dengue and Severe Dengue (SD) in the Americas, by Country. http://www.paho.org/hq/index.php?option=com_docman&task=doc_view&Itemid=&gid=32910&lang=en.

[6] PAHO (2021). Dengue Indicators. Dengue Serotypes by Year for Countries and Territories of the Americas. https://www3.paho.org/data/index.php/en/mnu-topics/indicadores-dengue-en/dengue-nacional-en/517-dengue-serotypes-en.html.

[7] Vaughn D.W., Green S., Kalayanarooj S., Innis B.L., Nimmannitya S., Suntayakorn S., Endy T.P., Raengsakulrach B., Rothman A.L., Ennis F.A. (2000). Dengue viremia titer, antibody response pattern, and virus serotype correlate with disease severity. J. Infect. Dis..

[8] Rico-Hesse R. (2003). Microevolution and virulence of dengue viruses. Adv. Virus Res..

[9] Green S., Rothman A. (2006). Immunopathological mechanisms in dengue and dengue hemorrhagic fever. Curr. Opin. Infect. Dis..

[10] Salles T.S., Sá-Guimarães T.D.E., De Alvarenga E.S.L., Guimarães-Ribeiro V., De Meneses M.D.F., De Castro-Salles P.F., Dos Santos C.R., Melo A.C.D.A., Soares M.R., Ferreira D.F. (2018). History, epidemiology and diagnostics of dengue in the American and Brazilian contexts: A review. Parasites Vectors.

[11] Wilson M.E., Chen L.H. (2002). Dengue in the Americas. Dengue Bull..

[12] Figueiredo R.M., Naveca F.G., Bastos M.S., Melo M.N., Viana S.S., Mourão M.P., Costa C.A., Farias I.P. (2008). Dengue virus type 4, Manaus, Brazil. Emerg. Infect. Dis..

[13] Dick O.B., Martín J.L.S., Del Diego J., Montoya R.H., Dayan G.H., Zambrano B. (2012). The history of dengue outbreaks in the Americas. Am. J. Trop. Med. Hyg..

[14] Andrioli D.C., Busato M.A., Lutinski J.A. (2020). Spatial and temporal distribution of dengue in Brazil, 1990–2017. PLoS ONE.

[15] Bezerra J.M.T., Sousa S.C.D., Tauil P.L., Carneiro M., Barbosa D.S. (2021). Entry of dengue virus serotypes and their geographic distribution in Brazilian federative units: A systematic review. Rev. Bras. Epidemiol..

[16] Figueiredo R.M.P.D., Mourão M.P.G., Abi-Abib Y.E.C., Oliveira C.M.D., Roque R., Azara T.D., Ohly J., Degener C., Geier M., Eiras Á.E. (2013). Identification of dengue viruses in naturally infected *Aedes aegypti* females captured with BioGents (BG)-Sentinel traps in Manaus, Amazonas, Brazil. Rev. Soc. Bras. Med. Trop..

[17] Franz A.W.E., Kantor A.M., Passarelli A.L., Clem R.J. (2015). Tissue barriers to arbovirus infection in mosquitoes. Viruses.

[18] Kramer L.D., Ciota A.T. (2015). Dissecting vectorial capacity for mosquito-borne viruses. Curr. Opin. Virol..

[19] Uno N., Ross T.M. (2018). Dengue virus and the host innate immune response. Emerg. Microbes Infect..

[20] Woodring J., Higgs S., Beaty B. (1996). Natural cycles of vector-borne pathogens. the Biology of Disease Vectors.

[21] Richards S.L., Anderson S.L., Alto B.W. (2012). Vector competence of *Aedes aegypti* and *Aedes albopictus* (Diptera: Culicidae) for dengue virus in the Florida Keys. J. Med. Entomol..

[22] Gonçalves C.M., Melo F.F., Bezerra J.M., Chaves B.A., Silva B.M., Silva L.D., Pessanha J.E., Arias J.R., Secundino N.F., Norris D.E. (2014). Distinct variation in vector competence among nine field populations of *Aedes aegypti* from a Brazilian dengue-endemic risk city. Parasittes Vectors.

[23] Poole-Smith B.K., Hemme R.R., Delorey M., Felix G., Gonzalez A.L., Amador M., Hunsperger E.A., Barrera R. (2015). Comparison of vector competence of *Aedes mediovittatus* and *Aedes aegypti* for dengue virus: Implications for dengue control in the Caribbean. PLoS Negl. Trop. Dis..

[24] Amoa-Bosompem M., Kobayashi D., Itokawa K., Murota K., Faizah A.N., Azerigyik F.A., Hayashi T., Ohashi M., Bonney J.H.K., Dadzie S. (2021). Determining vector competence of *Aedes aegypti* from Ghana in transmitting dengue virus serotypes 1 and 2. Parasites Vectors.

[25] Vazeille-falcoz M., Mousson L., Rodhain F., Chungue E., Failloux A.B. (1999). Variation in oral susceptibility to dengue type 2 virus of populatios of *Aedes aegypti* from the islands of Tahiti and Moorea, French Polynesia. Am. J. Trop. Med. Hyg..

[26] Bennett K.E., Farfan-Ale J.A., Fernandez-Salas I., Black W.C., Higgs S., Beaty B.J., Muñoz M.D.L., Olson K.E. (2002). Variation in vector competence for dengue 2 virus among 24 collections of *Aedes aegypti* from Mexico and the United States. Am. J. Trop. Med. Hyg..

[27] Godoy R.S.M., Felix L.d.S., Orfanó A.d.S., Chaves B.A., Nogueira P.M., Costa B.D.A., Soares A.S., Oliveira C.C.A., Nacif-Pimenta R., Silva B.M. (2021). Dengue and Zika virus infection patterns vary among *Aedes aegypti* field populations from Belo Horizonte, a Brazilian endemic city. PLoS Negl. Trop. Dis..

[28] Tabachnick W.J. (2013). Nature, nurture and evolution of intra-species variation in mosquito arbovirus transmission competence. Int. J. Environ. Res. Public Health.

[29] Failloux A.B., Vazeille M., Rodhain F. (2002). Geographic genetic variation in populations of the dengue virus vector *Aedes aegypti*. J. Mol. Evol..

[30] Faucon F., Dusfour I., Gaude T., Navratil V., Boyer F., Chandre F., Sirisopa P., Thanispong K., Juntarajumnong W., Poupardin R. (2015). Identifying genomic changes associated with insecticide resistance in the dengue mosquito *Aedes aegypti* by deep targeted sequencing. Genome Res..

[31] Bosio C.F., Fulton R.E., Salasek M.L., Beaty B.J., Black W.C. (2000). Quantitative trait loci that control vector competence for dengue-2 virus in the mosquito *Aedes aegypti*. Genetics.

[32] Fansiri T., Fontaine A., Diancourt L., Caro V., Thaisomboonsuk B., Richardson J.H., Jarman R.G., Ponlawat A., Lambrechts L. (2013). Genetic mapping of specific interactions between *Aedes aegypti* mosquitoes and dengue viruses. PLoS Genet..

[33] Smartt C.T., Shin D., Alto B.W. (2017). Dengue serotype-specific immune response in *Aedes aegypti* and *Aedes albopictus*. Mem. Inst. Oswaldo Cruz..

[34] Huber K., Le Loan L., Hoang T.H., Ravel S., Rodhain F., Failloux A.-B. (2002). Genetic differentiation of the dengue vector, *Aedes aegypti* (Ho Chi Minh City, Vietnam) using microsatellite markers. Mol. Ecol..

[35] Mousson L., Vazeille M., Chawprom S., Prajakwong S., Rodhain F., Failloux A.B. (2002). Genetic structure of *Aedes aegypti* populations in Chiang Mai (Thailand) and relation with dengue transmission. Trop. Med. Int. Health.

[36] Julio N.B., Chiappero M.B., Rossi H.J., Rondan Dueñas J.C., Gardenal C.N. (2009). Genetic structure of *Aedes aegypti* in the city of Córdoba (Argentina), a recently reinfested area. Mem. Inst. Oswaldo Cruz..

[37] Endersby N.M., Hoffmann A.A., White V.L., Ritchie S.A., Johnson P.H., Weeks A.R. (2011). Changes in the genetic structure of *Aedes aegypti* (Diptera: Culicidae) populations in Queensland, Australia, across two seasons: Implications for potential mosquito releases. J. Med. Entomol..

[38] Ayala A.M., Vera N.S., Chiappero M.B., Almirón W.R., Gardenal C.N. (2020). Urban populations of *Aedes aegypti* (Diptera: Culicidae) from central Argentina: Dispersal patterns assessed by Bayesian and multivariate Methods. J. Med. Entomol..

[39] Regilme M.A.F., Carvajal T.M., Honnen A.C., Amalin D.M., Watanabe K. (2021). The influence of roads on the fine-scale population genetic structure of the dengue vector *Aedes aegypti* (Linnaeus). PLoS Negl. Trop. Dis..

[40] Da Costa-Fraga E., dos Santos J.M., de Freitas-Maia J. (2003). Enzymatic variability in *Aedes aegypti* (Diptera: Culicidae) populations from Manaus-AM, Brazil. Genet. Mol. Biol..

[41] Santos J.M.M., Fraga E.C., Maia J.F., Tadei W.P. (2011). Genetic diversity in dengue mosquito, *Aedes aegypti* (Diptera: Culicidae) from Amazon Region: Comparative analysis with isozymes and RAPD Loci. Open Trop. Med. J..

[42] Instituto Brasileiro de Geografia e Estatística (IBGE) Cidades: Amazonas: Manaus. http://www.cidades.ibge.gov.br/.

[43] Reed L.J., Muench H. (1938). A simple method of estimating fifty percent endpoints. Am. J. Hyg..

[44] Secundino N.F.C., Chaves B.A., Orfano A.S., Silveira K.R.D., Rodrigues N.B., Campolina T.B., Nacif-Pimenta R., Villegas L.E.M., Silva B., Lacerda M.V.G. (2017). Zika virus transmission to mouse ear by mosquito bite: A laboratory model that replicates the natural transmission process. Parasites Vectors.

[45] Chan M., Johansson M.A. (2012). The Incubation Periods of Dengue Viruses. PLoS ONE.

[46] Kuno G., Chang G.-J.J., Tsuchiya K.R., Karabatsos N., Cropp C.B. (2009). Development and validation of real-time one-step reverse transcription-PCR for the detection and typing of dengue viruses. J. Clin. Virol..

[47] Villegas L.E., Campolina T.B., Barnabe N.R., Orfano A.S., Chaves B.A., Norris D.E., Pimenta P.F., Secundino N.F.C. (2018). Zika virus infection modulates the bacterial diversity associated with *Aedes aegypti* as revealed by metagenomic analysis. PLoS ONE.

[48] Bezerra J.M., Araújo R.G., Melo F.F., Goncalves C.M., Chaves B.A., Silva B.M., Silva L.D., Brandão S.T., Secundino N.F., Norris D.E. (2016). *Aedes (Stegomyia) albopictus*’ dynamics influenced by spatiotemporal characteristics in a Brazilian dengue-endemic risk city. Acta Trop..

[49] Morales-Vargas R.E., Missé D., Chavez I.F., Kittayapong P. (2020). Vector Competence for dengue-2 viruses isolated from patients with different disease severity. Pathogens.

[50] Pinheiro T.M., Mota M.T.D.O., Watanabe A.S.A., Biselli-Périco J.M., Drumond B.P., Ribeiro M.R., Vedovello D., Araújo J.P., Pimenta P.F.P., Chaves B.A. (2018). Viral immunogenicity determines epidemiological fitness in a cohort of DENV-1 infection in Brazil. PLoS Negl. Trop. Dis..

[51] Taracena M.L., Bottino-Rojas V., Talyuli O.A., Walter-Nuno A.B., Oliveira J.H.M., Angleró-Rodriguez Y.I., Wells M.B., Dimopoulos G., Oliveira P.L., Paiva-Silva G.O. (2018). Regulation of midgut cell proliferation impacts *Aedes aegypti* susceptibility to dengue virus. PLoS Negl. Trop. Dis..

[52] De Souza R.F. (2008). Mapeamento da incidência de dengue em Manaus (2008): Estudo da associação entre fatores socioambientais na perspectiva da Geografia da Saúde. Somanlu Revista Estudos Amazônicos.

[53] Oliveira F.N., Araújo R., Carvalho J., Costa S. (2008). Determination of variation in the Manaus-AM microclimate for anthropogenic activities and natural climatic modulations. Acta Amaz..

[54] Sarges R.R., Mendes T., Riccomini C. (2011). Caracterização do relevo da região de Manaus, Amazônia central. Rev. Bras. Geomorfol..

[55] Bell S.M., Katzelnick L., Bedford T. (2019). Dengue genetic divergence generates within-serotype antigenic variation, but serotypes dominate evolutionary dynamics. Elife.

[56] Bastos M.D.S., De Figueiredo R.M.P., Ramasawmy R., Itapirema E., Gimaque J.B.L., Santos L.O., Figueiredo L.T.M., Mourão M.P.G. (2012). Simultaneous circulation of all four dengue serotypes in Manaus, State of Amazonas, Brazil in 2011. Rev. Soc. Bras. Med. Trop..

[57] Ministério da Saúde (2021). Boletim epidemiológico 21. Monitoramento dos casos de arboviroses urbanas causados por vírus transmitidos pelo mosquito *Aedes* (dengue, chikungunya e zika), semanas epidemiológicas 1 a 21, 2021. Bol. Epidemiol..

[58] Chen R., Vasilakis N. (2011). Dengue—Quo tu et quo vadis?. Viruses.

[59] Kuno G., Chang G.J., Tsuchiya K.R., Karabatsos N., Cropp C.B. (1998). Phylogeny of the genus *Flavivirus*. J. Virol..

[60] Powell J.R. (2019). An evolutionary perspective on vector-borne diseases. Front. Genet..

[61] Danet L., Beauclair G., Berthet M., Moratorio G., Gracias S., Tangy F., Choumet V., Jouvenet N. (2019). Midgut barriers prevent the replication and dissemination of the yellow fever vaccine in *Aedes aegypti*. PLoS Negl. Trop. Dis..

[62] Khoo C.C.H., Piper J., Sanchez-Vargas I., Olson K.E., Franz A.W.E. (2010). The RNA interference pathway affects midgut infection- and escape barriers for Sindbis virus in *Aedes aegypti*. BMC Microbiol..

[63] Kramer L.D., Hardy J.L., Presser S.B., Houk E.J. (1981). Dissemination barriers for western equine encephalomyelitis virus in *Culex tarsalis* infected after ingestion of low viral doses. Am. J. Trop. Med. Hyg..

[64] Guzman M.G., Halstead S.B., Artsob H., Buchy P., Farrar J., Gubler D.J., Hunsperger E., Kroeger A., Margolis H.S., Martínez E. (2010). Dengue: A continuing global threat. Nat. Rev. Microbiol..

[65] Palmer W.H., Varghese F.S., Van Rij R.P. (2018). Natural variation in resistance to virus infection in dipteran insects. Viruses.

[66] Yasunaga A., Hanna S.L., Li J., Cho H., Rose P.P., Spiridigliozzi A., Gold B., Diamond M.S., Cherry S. (2014). Genome-wide RNAi screen identifies broadly-acting host factors that inhibit arbovirus infection. PLoS Pathog..

[67] Salazar M.I., Richardson J.H., Sánchez-Vargas I., Olson K.E., Beaty B.J. (2007). Dengue virus type 2: Replication and tropisms in orally infected *Aedes aegypti* mosquitoes. BMC Microbiol..

[68] Grunnill M., Boots M. (2015). How important is vertical transmission of dengue viruses by mosquitoes (Diptera: Culicidae)?. J. Med. Entomol..

[69] Regilme M.A.F., Carvajal T.M., Honnen A., Amalin D.M., Watanabe K. (2021). Brazilian *Aedes aegypti* as a competent vector for multiple complex arboviral coinfections. J. Infect. Dis..

[70] Girard Y.A., Klingler K.A., Higgs S. (2004). West Nile virus dissemination and tissue tropisms in orally infected *Culex pipiens quinquefasciatus*. Vector Borne Zoonotic Dis..

[71] Weaver S.C. (1986). Electron microscopic analysis of infection patterns for Venezuelan equine encephalomyelitis virus in the vector mosquito, *Culex (Melanoconion) taeniopus*. Am. J. Trop. Med. Hyg..

[72] da Moura A.J.F., de Melo Santos M.A.V., Oliveira C.M.F., Guedes D.R.D., de Carvalho-Leandro D., da Cruz Brito M.L., Rocha H.D.R., Gómez L.F., Ayres C.F.J. (2015). Vector competence of the *Aedes aegypti* population from Santiago Island, Cape Verde, to different serotypes of dengue virus. Parasites Vectors.

